# Case report: an unrecognized etiology of transient gallbladder pain in heart failure diagnosed with internist-performed point-of-care ultrasound

**DOI:** 10.1186/s13089-014-0019-8

**Published:** 2015-01-21

**Authors:** Christine N Desautels, David M Tierney, Federico Rossi, Terry K Rosborough

**Affiliations:** Department of Medical Education, Abbott Northwestern Hospital, Graduate Medical Education, Mail Route #11135, 800 E. 28th Street, Minneapolis, MN 55407 USA; Minnesota Gastroenterology, 15700 37th Avenue North #300, Plymouth, MN 55446 USA

**Keywords:** Point-of-care ultrasound, Heart failure, Gallbladder, Cholecystitis, Cholecystalgia

## Abstract

The excellent sensitivity and specificity of right upper quadrant (RUQ) ultrasound for gallbladder pathology in patients with abdominal pain is heavily relied upon in routine diagnostic evaluation. The hour-to-hour timing of this test in a patient with fluctuating symptoms is not widely recognized as having a significant impact on its sensitivity. However, we present a case report describing the essential role of symptom-timed point-of-care ultrasound in making an elusive diagnosis of transient cholecystalgia in a patient with RUQ pain and congestive heart failure (CHF). This case also demonstrates an important etiology of RUQ pain in patients with CHF beyond that of congestive hepatopathy. A review of the related entities of acalculous cholecystitis, congestive hepatopathy, and diffuse gallbladder wall thickening is provided.

## Case presentation

A 47-year-old Ethiopian woman with a history of rheumatic heart disease resulting in severe mitral and tricuspid regurgitation, pulmonary hypertension, congestive heart failure, and atrial fibrillation presented with 3 days of cough, increased shortness of breath, and myalgias. Vital signs on admission were notable for a temperature of 99.7 **°**F and tachycardia (heart rate: 110 to 120 bpm). Examination findings included the expected cardiac murmurs and mild expiratory wheeze. EKG showed atrial fibrillation. Laboratory evaluation was notable for an unremarkable complete blood count and basic metabolic panel, normal troponin I, and mildly elevated brain natriuretic peptide of 296 pg/mL (normal range <150 pg/mL). Chest X-ray showed cardiomegaly with no acute infiltrate.

**Figure 1:**
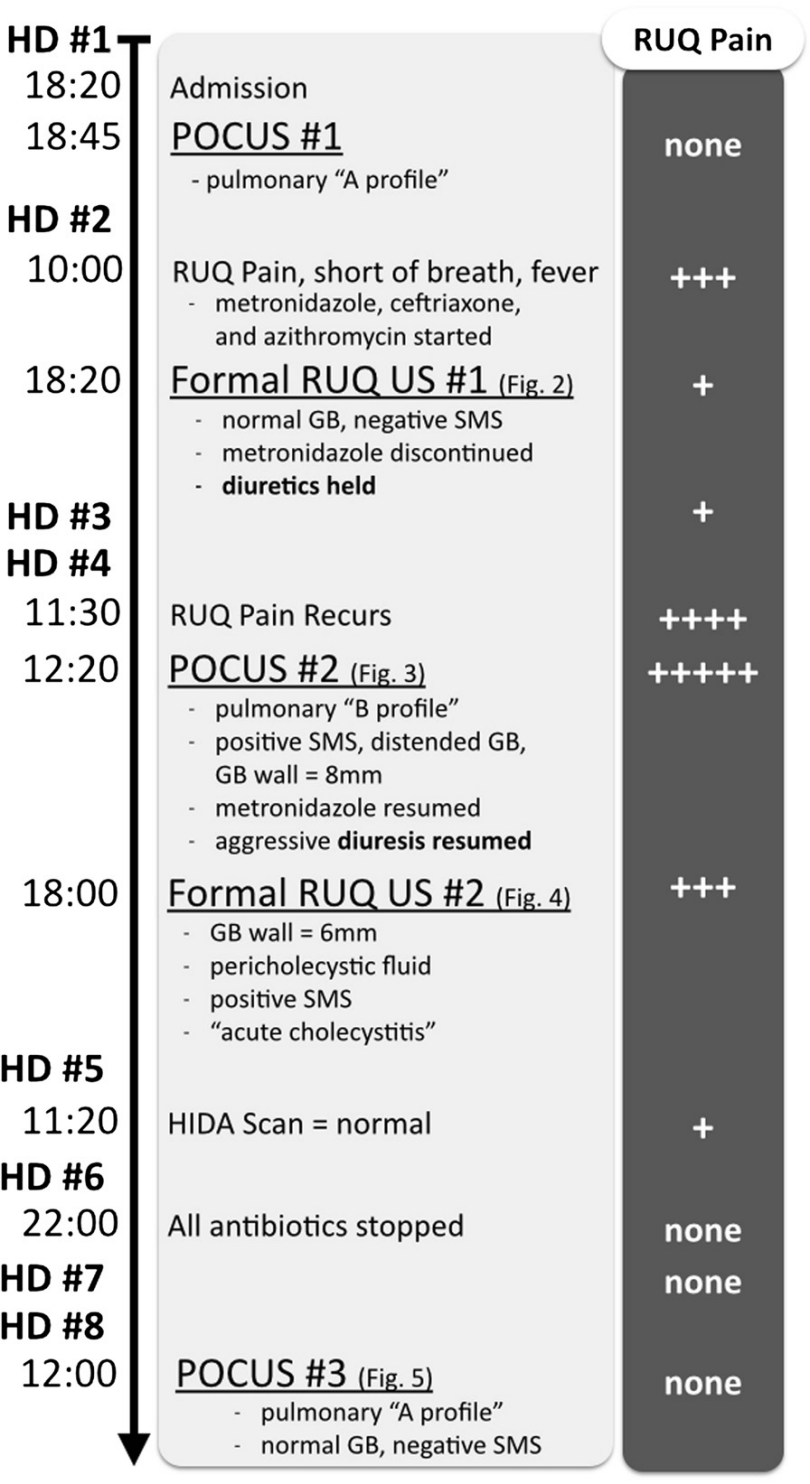
**Timeline of hospital events and severity of right upper quadrant pain.** HD, hospital day; POCUS, point-of-care ultrasound; RUQ, right upper quadrant; US, ultrasound; GB, gallbladder; SMS, sonographic Murphy sign. The cross symbol represents the patient’s pain level.

A point-of-care ultrasound (POCUS) exam (Figure 1, Timeline = ‘POCUS #1’) was performed by the internal medicine resident with an ultrasound mentor and showed a bilateral pulmonary A-profile (normal) without an interstitial process (e.g., cardiogenic pulmonary edema) or consolidation, normal left ventricular systolic function, massive biatrial enlargement, severe mitral and tricuspid regurgitation, a small pericardial effusion, and a 2.4-cm inferior vena cava with 10% inspiratory collapse. A diagnosis of influenza was initially suspected given the community prevalence and the patient's residence in a shelter where others had been ill. Empiric treatment with oseltamivir was initiated. However, rapid influenza swab and subsequent PCR were both negative.

On hospital day 2, the patient had fever to 101 **°**F and complained of increasing right upper quadrant (RUQ) abdominal pain. Laboratory testing revealed an elevated bilirubin of 1.5 mg/dL (0.6 mg/dL direct component) and alkaline phosphatase of 146 IU/L (normal range <136 IU/L). Aminotransferase levels were normal. It was noted that the patient had been admitted 1 month prior with RUQ abdominal pain and vomiting at which time a formal diagnostic ultrasound showed gallstones, a thickened gallbladder wall (8 mm), and pericholecystic fluid consistent with acute cholecystitis. General surgery and gastroenterology had recommended cholecystectomy after her congestive heart failure had been medically and/or surgically optimized, which had not yet occurred. The differential for her fever was thus broadened from solely a pulmonary source to include acute cholecystitis and cholangitis. Broad antibiotic therapy was added with metronidazole, ceftriaxone, and azithromycin for coverage of pneumonia as well as a possible biliary etiology. Diuretics were held due to hypotension and concern for early sepsis.

**Figure 2:**
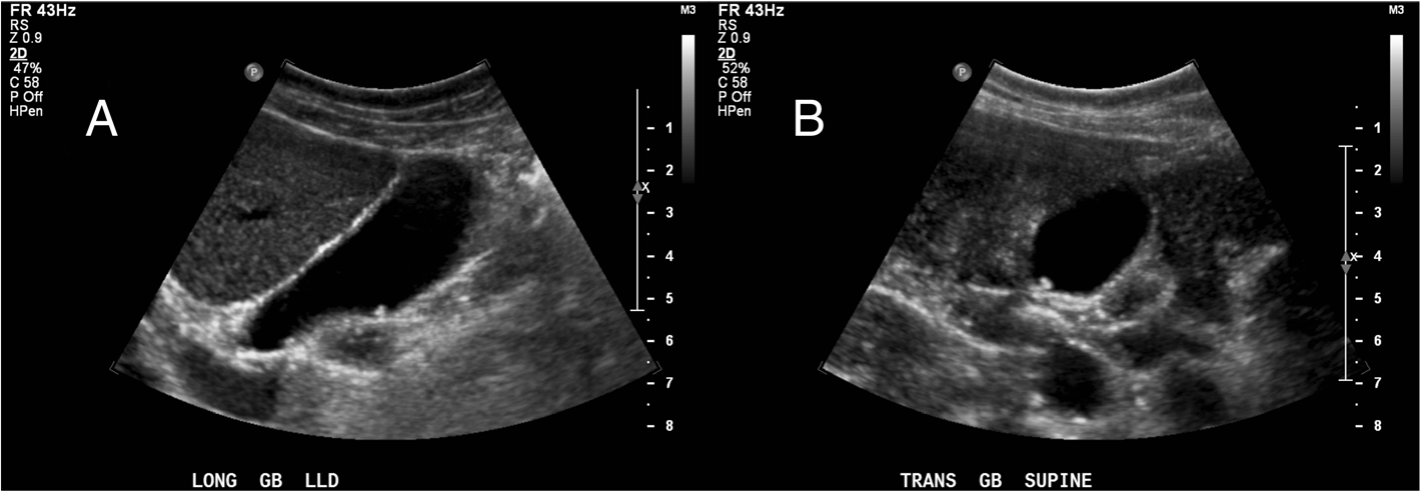
**Formal right upper quadrant ultrasound #1. (A)** Long and **(B)** short axis views showing a few small stones within the gallbladder, with no gallbladder wall thickening or pericholecystic fluid.

A formal RUQ ultrasound (Figure 1, Timeline = ‘Formal RUQ US #1’) was obtained that afternoon, 8 h after bedside assessment of the patient, and showed a few small stones within the gallbladder, a normal gallbladder wall, no pericholecystic fluid, no ductal dilatation (Figure 2), and a negative sonographic Murphy sign. Given this imaging result, metronidazole was discontinued and alternative etiologies for abdominal pain were added back to the differential.

**Figure 3:**
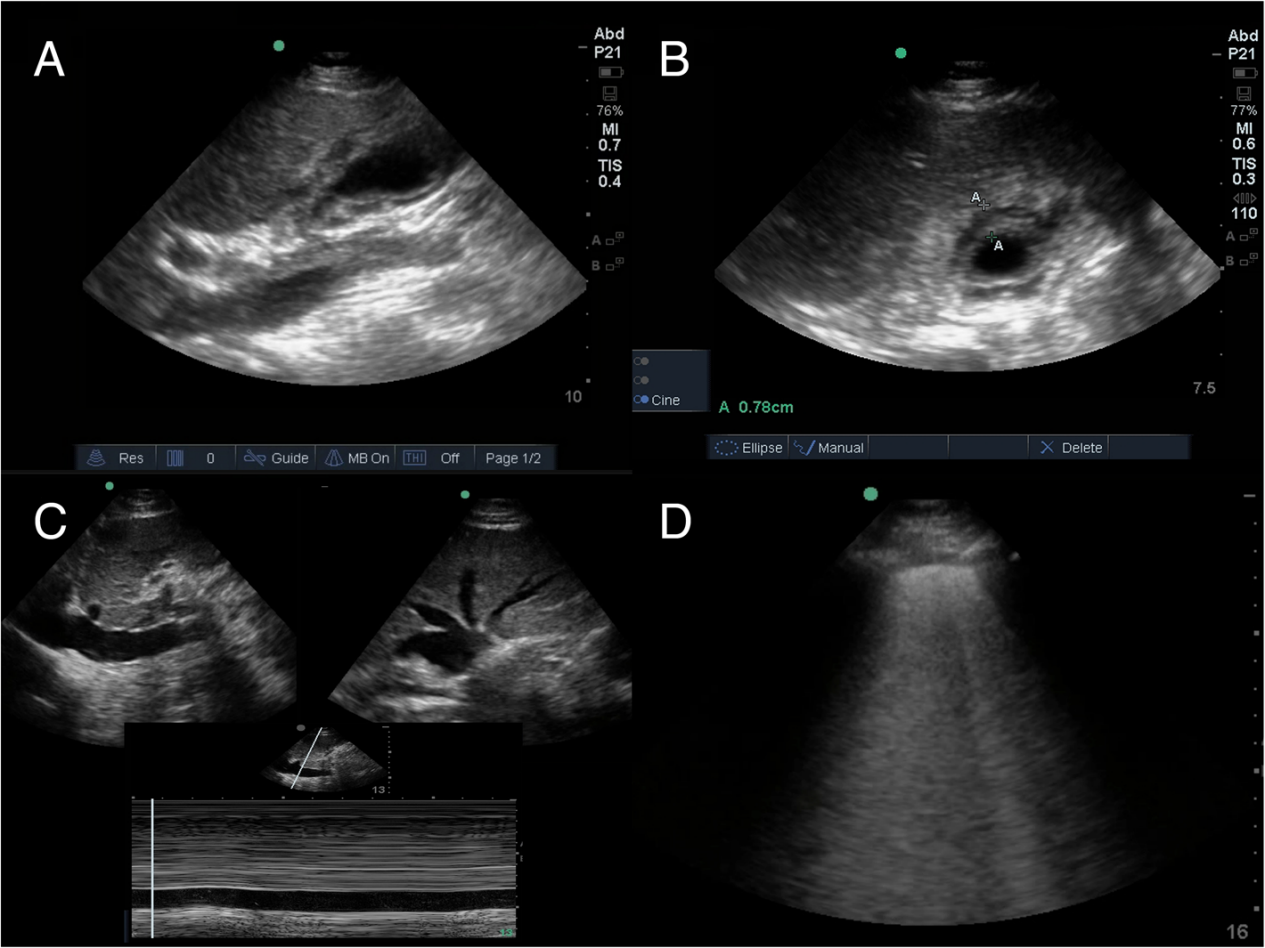
**POCUS #2. (A)** Long and **(B)** short axis views of the gallbladder showing abnormal gallbladder wall thickening to 0.78 cm with edematous narrowing of the gallbladder neck. **(C)** IVC measuring 2 cm with <10% inspiratory collapse and associated hepatic vein engorgement indicating volume overload. **(D)** Diffuse pulmonary B-lines consistent with interstitial edema.

On hospital day 4, the patient complained of recurrent, severe RUQ and epigastric pain. Exam revealed severe tenderness to palpation in the RUQ and voluntary guarding, a significant change from the previous day. A POCUS exam (Figure 1, Timeline = ‘POCUS #2’) was repeated by the internal medicine resident with an ultrasound mentor and showed bilateral diffuse pulmonary B-lines (interstitial pattern) consistent with new pulmonary edema. Despite the unremarkable formal RUQ ultrasound 42 h prior, POCUS at the time of her recurrent RUQ pain revealed a markedly abnormal gallbladder with wall thickening to 8 mm, narrowing of the gallbladder neck due to edema, a small non-obstructing gallstone, a normal common bile duct, subserosal edema, and pericholecystic fluid (Figure 3). A positive sonographic Murphy sign was demonstrated when the patient was asked to ‘push with the ultrasound probe and find where it hurts most’ and the probe was placed directly over the gallbladder fundus.

**Figure 4:**
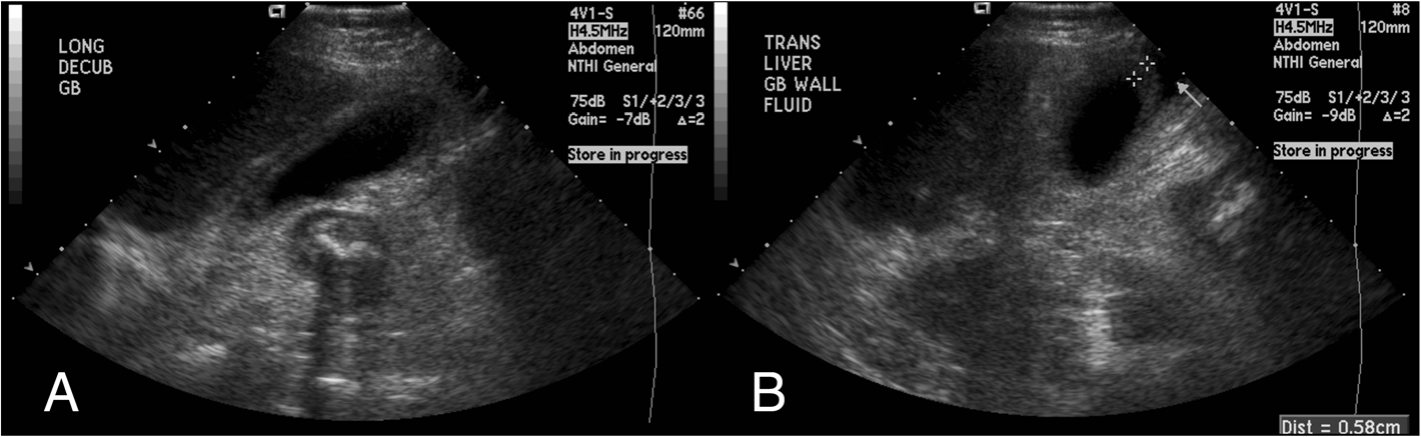
**Formal right upper quadrant ultrasound #2. (A)** Formal ultrasound long axis and **(B)** short axis views confirming gallbladder wall thickening (0.58 cm, 6 h after bedside ultrasound and initiation of diuresis).

Metronidazole was resumed, and general surgery and gastroenterology were consulted to assist in the management of acute cholecystitis. Diuresis was resumed in light of the POCUS findings supporting new pulmonary edema. A repeat formal RUQ ultrasound (6 h later) (Figure 1, Timeline = ‘Formal RUQ US #2’) confirmed new marked echogenic gallbladder wall thickening (decreased to 6 mm with diuresis between POCUS and the formal study), pericholecystic fluid, and a normal common bile duct with no impacting cholelithiasis (Figure 4). The formal RUQ ultrasound impression was ‘acute cholecystitis.’ Cholecystectomy versus percutaneous cholecystostomy was recommended, pending the outcome of cholescintigraphy and the patient's overall medical stability.

**Figure 5:**
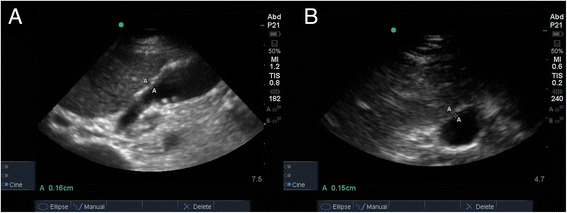
**POCUS #3. (A)** Long axis and **(B)** short axis views showing improvement in gallbladder wall thickening after diuresis, with gallbladder wall thickness measuring 0.15 cm.

Cholescintigraphy was obtained on hospital day 5 following significant diuresis and was negative for acute cholecystitis. Given this, and the fact that the patient was improving and laboratory abnormalities had normalized, gastroenterology and general surgery recommended ongoing conservative management. Attention turned toward optimization of the patient's congestive heart failure as the cause of secondary cholecystalgia. Diuresis was increased on hospital day 7. By the following day, the patient's weight had decreased by 2.8 kg; she was feeling much improved and exam revealed only mild, vague right upper quadrant tenderness. POCUS exam (Figure 1, Timeline = ‘POCUS #3’) was again repeated and showed significant improvement, without gallbladder wall thickening or sonographic Murphy sign (Figure 5). The patient was discharged without RUQ pain after optimization of her volume status *without* plans for future cholecystectomy.

## Background

The initial differential diagnosis for this patient's right upper quadrant pain included acute gallstone-mediated cholecystitis, acalculous cholecystitis, and congestive hepatopathy. The entity of transient cholecystalgia, fluctuating in real time with volume status, was not initially considered. This led to unnecessary testing for alternative etiologies, plans for cholecystectomy, and significant confusion for the patient and providers when the initial formal RUQ ultrasound did not corroborate the bedside physical exam and point-of-care ultrasound. A brief review of the considered etiologies of RUQ pain and discussion of the appropriate weight we should place on our clinical and imaging diagnostic tests for the gallbladder follows.

### Accuracy of gallbladder diagnostics

The right upper quadrant ultrasound is the most commonly used imaging modality in the evaluation of gallbladder disease. The test's accuracy for acute cholecystitis has been debated due to the presence of verification bias [1,2]. A meta-analysis of ultrasound's accuracy for acute cholecystitis found a sensitivity of 94% and specificity of 78%. When the results were adjusted for verification bias, the sensitivity decreased to 88% and specificity increased to 80%. The authors recommended using estimates between the adjusted and unadjusted values. Radionucleotide scanning has better sensitivity (97%) and specificity (90%) for acute cholecystitis [1].

We rely heavily on these test characteristics of formal ultrasound because our clinical and laboratory testing alone do not have the power to rule in or out the diagnosis of acute cholecystitis. The negative likelihood ratio (LR) of RUQ tenderness is 0.4 (95% confidence interval 0.2 to 1.1), representing the most helpful clinical finding in ruling out acute cholecystitis. However, lack of RUQ tenderness is probably weaker than this for excluding acute cholecystitis when studies are adjusted for verification and spectrum bias. The positive LR of the Murphy sign at 2.8 (0.8 to 8.6) is the most helpful finding for strengthening a diagnosis of acute cholecystitis and appears to be even stronger when the data are adjusted for verification and spectrum bias. Notably, the 95% confidence intervals for both of these likelihood ratios cross 1. The ‘clinical impression’ of cholecystitis, which includes an unspecified combination of history, physical exam, and laboratory findings, results in the best test with an estimated positive LR of 25 to 30 [2].

In the case of our patient, the presence of gallstones, gallbladder wall thickening, and a positive sonographic Murphy sign has extremely high positive predictive value (98.5%) for a gallbladder requiring surgical intervention [3]. However, she did not require cholecystectomy once the secondary nature of her gallbladder abnormality was confirmed.

### Acalculous cholecystitis

Acute acalculous cholecystitis (ACC) remains a menacing diagnosis that carries a mortality rate of at least 30% [4]. Though traditionally associated with critical illness, trauma, burns, and the post-operative period, ACC can occur in a variety of other scenarios. It has been hypothesized that major contributing factors include bile stasis and gallbladder ischemia, followed by secondary infection with enteric pathogens. Bile stasis has been attributed to volume depletion, opioid-induced sphincter of Oddi spasm, gastrointestinal hypomotility, and positive-pressure mechanical ventilation. Gallbladder ischemia may be due to systemic hypotension, possibly exacerbated by increased intraluminal pressure from bile stasis, which together decrease the gallbladder perfusion pressure [5,6]. ACC can occur in the setting of congestive heart failure (CHF), where the gallbladder perfusion pressure presumably suffers due to decreased cardiac output and elevated venous pressures [4,7,8]. Unlike the scenario presented in our case, ACC is typically not transient.

The pathogenesis of ACC involves inflammation and necrosis of the gallbladder in the absence of an obstructing gallstone. Signs and symptoms include fever, anorexia, nausea, vomiting, RUQ or upper abdominal tenderness, and Murphy sign (if the patient is able to communicate pain). Laboratory abnormalities include leukocytosis and elevated alkaline phosphatase, bilirubin, and aminotransferases. Unfortunately, none of these findings are particularly sensitive or specific in the affected patient population, and diagnosis relies heavily on the physician's assessment of the overall clinical picture [2,7].

Ultrasonography is the preferred test for diagnosis due to the typical location of these patients in the intensive care unit; however, the test has a sensitivity ranging from 30% to 100% [6]. Radionucleotide scanning, more cumbersome in sick patients and fraught with false positives, has better sensitivity for ACC with values ranging from 67% to 100% [6] and a review targeting a sensitivity of 91% [9]. The treatment is surgical, with cholecystectomy or percutaneous cholecystostomy in addition to antibiotic therapy.

### Congestive hepatopathy

Congestive hepatopathy, a common diagnosis in patients with decompensated heart failure and RUQ pain, is a well-established clinical syndrome that can result from any cause of right heart failure. Elevated central venous pressure in congestive heart failure is transmitted to the hepatic veins and to the venules draining hepatic acini, and these increased pressures can result in atrophy of hepatocytes and perisinusoidal edema that impairs diffusion of oxygen and nutrients [10,11].

Patients with hepatic congestion are usually asymptomatic but can present with RUQ pain attributed to stretching of the liver capsule, usually seen during acute exacerbations of congestive heart failure [12]. The most common laboratory abnormalities attributed to congestive hepatopathy in patients with chronic CHF are decreased albumin, elevated serum bilirubin, and elevated alkaline phosphatase [13]. Generally, when abnormalities are present, they are mild: serum bilirubin is typically less than 3 mg/dL, alkaline phosphatase is typically normal or only minimally elevated, and aminotransferases are usually normal and rarely greater than two times the upper limit of normal [14,15]. However, there is some evidence that in more severe congestive heart failure (on the basis of cardiac index ≤1.5 L/min/m^2^), more significant abnormalities in liver function tests are seen, including aminotransferase elevations >400 U/L [15]. One study found that elevated serum bilirubin was an independent predictor of adverse cardiovascular outcomes and all-cause mortality in patients with chronic CHF [13].

The typical ultrasound findings in congestive hepatopathy are non-specific and can include a dilated, non-collapsible inferior vena cava, hepatomegaly, ascites, and Doppler waveform abnormalities in the portal veins. The right upper quadrant ultrasound is primarily utilized to rule out other causes of RUQ pain [16,17].

Once identified, management of congestive hepatopathy primarily involves treatment of the underlying heart disease, including careful diuresis and efforts to avoid decreasing cardiac output.

### Diffuse gallbladder wall thickening

While heart failure can cause congestive hepatopathy, it is also known to be a cause of diffuse gallbladder wall thickening. This finding is non-specific and can occur in many other states including acute cholecystitis, cirrhosis, hypoalbuminemia, adenomyomatosis, malignancy, hepatitis, pancreatitis, etc. The diffuse wall thickening related to elevated portal and systemic venous pressures such as in cirrhosis and heart failure is typically *not* thought to cause pain and a positive Murphy sign [18–21]. In our patient, a definite focal Murphy sign was present at the time of the wall thickening (and pain symptoms) and resolved with resolution of wall thickening.

## Conclusions

This case describes an important additional diagnosis to consider for the common clinical presentation of RUQ pain in a patient with congestive heart failure. Specifically, it illustrates the under-recognized entity of transient secondary cholecystalgia (gallbladder wall thickening and sonographic Murphy sign) fluctuating hour-by-hour with the patient's right-sided filling pressures.

This was a challenging diagnostic dilemma made more complicated by the discordance between the physicians' bedside clinical assessment and the available imaging results. RUQ abdominal pain, fever, nausea, and laboratory abnormalities all suggested the gallbladder as the source of the patient's pain. When the result of the formal RUQ ultrasound was unexpectedly normal, the clinical team found themselves reconsidering their diagnosis because of their confidence in the test's excellent sensitivity. However, the availability of point-of-care ultrasound allowed physicians familiar with the clinical scenario to obtain real-time imaging when symptoms suggestive of gallbladder pathology recurred within hours of the normal formal RUQ ultrasound, maximizing the sensitivity of the test and subsequently avoiding the cost and risk of further diagnostic evaluation.

Once abnormal gallbladder wall thickening and positive sonographic Murphy sign were identified, the physicians initially suspected acalculous cholecystitis. This would have represented an atypical presentation of the diagnosis as the patient was neither critically ill nor recovering from surgery; though, as noted above, ACC can occur secondary to CHF, medications, *nil per os* status, or other systemic illness. However, the ability to perform follow-up POCUS exams allowed the clinical team to visualize rapid resolution of gallbladder wall thickening correlating in real time with diuresis. This confirmed a secondary cause of the gallbladder findings and resulted in a non-surgical intervention.

The possibility that the etiology of this patient's RUQ pain was actually infectious cholecystitis and that cessation of metronidazole resulted in the increased pain on hospital day 4 (Figure 1, Timeline = ‘HD #4, 11:30’) is unlikely. Metronidazole was held for at least four times its half-life; thus, symptoms probably would have recurred sooner if this were infectious cholecystitis. Additionally, though RUQ abdominal pain recurred, the patient did not have recurrence of fever after hospital day 2. Most convincingly, with adequate control of her volume status and in the absence of antibiotic therapy as an outpatient, our patient has had no evidence of cholecystitis.

While diffuse gallbladder wall edema is a known entity in heart failure, this case demonstrates that it is not always painless, and a positive sonographic Murphy sign can be present. Thus, the specificity of the Murphy sign for acute surgical cholecystitis, even when seen with wall thickening and in the presence of gallstones, should be cautiously applied in this patient population at generally higher surgical risk. We suspect that the presence of maximal pain directly over the gallbladder in this case was due in part to obstruction of the gallbladder neck from significant subserosal edema.

In conclusion, we have presented an illustrative case of gallbladder wall thickening and what clinically appeared to be cholecystitis fluctuating in real time with a patient's volume status and captured by point-of-care ultrasound. This entity likely represents a portion of the cases of right upper quadrant abdominal pain in the setting of CHF previously attributed to hepatic capsular distention. The pathophysiology is likely the same as in congestive hepatopathy and treatment in both cases should be directed at the underlying problem of volume overload. More importantly, this entity may also represent a subset of patients with CHF, bystander gallstones, and a thickened gallbladder wall who receive potentially unnecessary surgical treatment for presumed acute gallstone cholecystitis, as almost happened with our patient and as we have subsequently seen in a separate case.

In scenarios such as this, when imaging findings vary over hours with changes in patient physiology, the established sensitivity of formal imaging studies may no longer apply and over-reliance on a negative study can easily misdirect the differential diagnosis and treatment plan. Point-of-care ultrasound in the hands of a competent physician caring for the patient, obtained and interpreted in a known clinical setting, and repeated as necessary during fluctuations in patient status provides an invaluable tool for the complex problem solving frequently encountered by internal medicine physicians.

## Consent

Written informed consent was obtained from the patient for publication of this case report and any accompanying images. A copy of the written consent is available for review by the Editor-in-Chief of this journal.
